# Intraspecific Genomic Divergence and Minor Structural Variations in *Leishmania (Viannia) panamensis*

**DOI:** 10.3390/genes11030252

**Published:** 2020-02-27

**Authors:** Luz H. Patino, Marina Muñoz, Carlos Muskus, Claudia Méndez, Juan David Ramírez

**Affiliations:** 1 Grupo de Investigaciones Microbiológicas-UR (GIMUR), Departamento de Biología, Facultad de Ciencias Naturales, Universidad del Rosario, Bogotá 110211, Colombia; luzh.patino@urosario.edu.co (L.H.P.); claudiamarina23@gmail.com (M.M.); 2 Programa de Estudio y Control de Enfermedades Tropicales (PECET), Facultad de Medicina, Universidad de Antioquia, Medellín 50037, Colombia; carlos.muskus@udea.edu.co; 3 Dirección de Sanidad Militar, Ejercito Nacional de Colombia, Bogotá 110211, Colombia; claudia.mendez@ejercito.mil.co

**Keywords:** phylogenetic analysis, cutaneous leishmaniasis, DNA-seq, genomic variability, copy number variations, single-nucleotide polymorphisms, *Leishmania panamensis*

## Abstract

*Leishmania (Viannia) panamensis* is one of the most important *Leishmania* species associated with cutaneous leishmaniasis (CL) in Latin America. Despite its wide geographic distribution and pathogenic potential in humans and animals, the genomic variability of this species is low compared with other *Leishmania* species circulating in the same geographical area. No studies have reported a detailed analysis of the whole genome of *L. panamensis* from clinical isolates using DNA high-throughput sequencing to clarify its intraspecific genomic variability or plausible divergence. Therefore, this study aimed to evaluate the intraspecific genomic variability of *L. panamensis* from Colombia and Panama. A total of 22 genomes were analyzed, 19 from Colombian patients with CL and three genomes from Panama obtained from public databases. The phylogenomic analysis revealed the potential existence of three well-supported clades as evidence of intraspecific divergence. Additionally, the whole-genome analysis showed low structural variations in terms of ploidy, copy number variations, and single-nucleotide polymorphisms (SNPs). SNPs shared among all clades were identified, revealing their importance in different biological processes of *L. panamensis*. The findings not only expand our knowledge of intraspecific genomic variability of one of the most important *Leishmania* species in South America but also highlights the possible existence of different clades/lineages/subpopulations across a geographic scale.

## 1. Introduction

Cutaneous leishmaniasis (CL) is an endemic parasitic disease observed in several areas of the world. This clinical manifestation is widely distributed in regions such as sub-Saharan Africa, the Mediterranean, the Middle East, Central and South Asia, and Central and South America [1,2,3]. It can be caused by a wide variety of *Leishmania* species such as *L. braziliensis*, *L. amazonensis*, *L. aethiopica*, *L. mexicana*, *L. guyanensis*, *L. panamensis*, *L. peruviana*, *L. tropica*, and L. major [4]. Despite a large number of Leishmania species associated with this disease in South America, the main species responsible for CL are *L. panamensis* and *L. braziliensis* [5,6]. These species are characterized not only by their wide geographical distributions across the region and their associations with the intra- and peridomiciliary transmission cycles [5] but also by their abilities to infect different mammalian hosts and adapt to new vectors (sandflies). These eco-epidemiological features contribute to the wide genomic variability of this species; in addition, some studies have reported an association between this variability and the different clinical forms of the disease [7,8]. 

Several nuclear and mitochondrial DNA markers (cytochrome b, COII, gp63, 18S-rRNA, mini-exon, HSP-70, and ribosomal internal transcribed spacer regions (ITS-rDNA)) as well as different molecular techniques such as polymerase chain reaction (PCR) [9], multilocus sequence typing (MLST) [10], multilocus enzyme electrophoresis (MLEE) [5,11], random amplified polymorphism (RAPD) [12], amplified fragment length polymorphisms (AFLP) [13] and microsatellites [14], have been used to evaluate the inter and intraspecific genetic variability of different *Leishmania* species (*L. tropica*, *L. major*, *L. donovani*, *L. braziliensis*, *L. peruviana*, *L. guyanensis*, and *L. panamensis*) [13,14,15,16,17,18,19]. However, the identification of such genetic variants across the whole genome has become possible only with the arrival of DNA-seq. To date, high-throughput genomic sequencing has allowed detailed analysis of genomic variability between different species and strains [20], among closely related species of the same complex (*L. braziliensis/L. peruviana*) [21], and within the clinical isolates of the same species with high genomic similarity (e.g., *L. donovani* and *L. major*) [22,23]. The findings of previous studies have revealed that the genomic plasticity of this parasite allows structural and timely changes (chromosome and gene copy number variations (CNVs), single-nucleotide polymorphisms (SNPs), and insertions and deletions (Indels)) as a mechanism of rapidly adapting to different environments, stress conditions, and pressures in the hosts [24,25]. 

However, despite the number of studies reported to date, only few studies using the above-mentioned approaches have focused on evaluating such genomic variability in the clinical isolates of *Leishmania* species from the subgenus *Viannia*, such as the published study by de Figueiredo de Sá et al. who highlighted the high genetic diversity of *L. braziliensis* within restricted or adjacent areas [26], the study published by Urrea et al. who evaluated the genomic variations of four *L. panamensis* strains exhibiting different levels of virulence [27], and the recently published study by Restrepo et al. who described the genetic variations occurred in two *L. panamensis* clinical samples [28].

Although these studies have advanced our understanding of the genetic heterogeneity of *Leishmania* (*Viannia*) parasites, the information available for *L. panamensis* is scarce. This is particularly surprising considering the clinical and epidemiological importance of this species in some parts of South America, particularly Colombia and Panama. Therefore, the objective of the present study was to evaluate the phylogenetic relationships of 19 *L. panamensis* Colombian clinical isolates including a comparative approach with two publicly available genomes from Panama. This set of genomes was used to particularly focus on the large-scale screening of phylogenomics, ploidy changes, structural variations, and single point mutations.

## 2. Materials and Methods

### 2.1. Ethics Statement

This study was approved by the Ethics Committee of the Universidad de Antioquia (number VRI3445/2010) and by the Board of Ethical Conduct of Hospital Militar Central-Bogota, Colombia (HOMICE) in accordance with the resolutions number 36836 and 2043. Written informed consent was obtained from the patients from which the strains were isolated.

### 2.2. Study population

A total of 22 whole genomes were analyzed, including 19 from Colombian clinical isolates from patients with CL attending the ‘Programa de Estudio y Control de Enfermedades Tropicales (PECET), Medellín Colombia’, and ‘Dirección de Sanidad Militar, Ejercito Nacional de Colombia, Bogotá, Colombia’. Secondly, two publicly available Panamanian genomes from DDBJ/ENA/GenBank database (http://www.ebi.ac.uk/ena) under Run accession SRR10246848, SRR10246848 and one from TriTrypDB (http://tritrypdb.org: LpanamensisMHOMPA94PSC1) (LPPSC-1), were included for comparative purposes. 

### 2.3. Culture Conditions, DNA Extraction, and Species Identification

The 19 Colombian clinical isolates were axenically maintained in a Schneider medium supplemented with 10% (*v*/*v*) fetal bovine serum, and the isolates were cultured at 26 °C with 5% CO_2_. Approximately 1 × 10^6^ promastigotes in the late logarithmic growth phase were cultured and harvested by centrifugation for subsequent DNA extraction, which was conducted using the High Pure PCR Template Preparation Kit (Roche Life Science, Mannheim, Germany), in accordance with the manufacturer’s instructions. The DNA concentrations were determined with the NanoDrop ND-1000 spectrophotometer (Thermo Fisher Scientific Inc., Waltham, MA, USA), and the DNA quality and integrity were determined by 1% agarose gel electrophoresis. All the samples had an A_260_/A_280_ ratio of >2. Once the quality of DNA was verified, each sample was divided into two groups: one for species identification and the other for whole-genome sequencing.

Species identification was performed using the direct Sanger sequencing of genes encoding cytochrome b molecules and heat shock protein (HSP70), as described previously [5,6]. The amplification products were purified with EXOSAP (Affymetrix, Santa Clara, CA, USA) and sequenced using the dideoxy-terminal method in an automated capillary sequencer (AB3730; Applied Biosystem, Foster City, CA, USA). Subsequently, the sequences were subjected to BLASTn for a similarity search with the *Leishmania* sequences deposited in GenBank [5].

### 2.4. Genomic Sequencing and Data Analysis

The extracted whole-genome DNA was sequenced on a HiSeq X-Ten system (Illumina; Novogene Bioinformatics Technology Co., Ltd., Beijing, China). Briefly, mate-paired libraries were constructed by end repair (350-bp insert size) and subjected to paired-end sequencing (2 × 150-bp read length). Paired reads were discarded when reads with adapter contamination, >10% uncertain nucleotides, or >50% low-quality nucleotides (base quality <5) were identified [29].

### 2.5. DNA Mapping

The paired-end Illumina reads of 19 Colombian clinical isolates and of the three Panamanian genomes (data downloaded from DDBJ/ENA/GenBank (http://www.ebi.ac.uk/ena) and TriTrypDB (http://tritrypdb.org) databases) were mapped to the reference UA946 *L. panamensis* genome [27] (this genome was used as reference due to its high quality of assembly and annotation as reported elsewhere [27]) and assembled with the SMALT program (version 0.7.4) (www.sanger.ac.uk/resources/software/smalt/). The mapping involved the following parameters: exhaustive search option (−x and −y of 0.8), a reference hash index of 13 bases, and a sliding step of 3. An identity threshold of y = 0.8 prevented the mapping of non-*Leishmania* reads to the reference sequences because SMALT can trim reads before mapping them to the reference sequence. The read file merging, sorting, and elimination of PCR duplicates was implemented with SAMtools (version 0.1.18) and Picard (version 1.85) [30]. 

### 2.6. Nuclear and Mitochondrial Phylogenomic Inferences

The SNPs from whole nuclear and mitochondrial genomes were extracted and then used to build alignments. The preliminary clustering among *L. panamensis* isolates was explored using an approximately-maximum-likelihood phylogenetic tree built in FastTree double-precision version 2.1.10 [31]. The robustness of the nodes was evaluated using the bootstrap method (BT, with 1000 replicates). Phylogeographic relationships were comprehensively analyzed from each alignment using a Bayesian evolutionary approach based on Markov Chain Monte Carlo (MCMC) implemented in BEAST-2 [32], considering a node dating step using the geographic origin as reference metadata. For that, the best substitution model was initially chosen in jModelTest v0.1.1 [33]. The MCMC was then carried out considering a strict clock model and the Bayesian skyline population model, with a chain length of 2,000,000 states and resampling every 10% of the states. The effective sample size (ESS) was determined when ESS values were >200 for all parameters, indicating sufficient sampling, monitoring this, Bayesian Skylide analysis conducted in Tracer v1.7.1 [34]. Tree files were summarized with LogCombiner v1.10.4 (with burn-in of 300,000) and then were annotated in Tree Annotator v2.4.8 (with a burn-in of 100,000) [32], with maximum clade credibility and mean node heights. The obtained tree was visualized using the interactive tool Interactive Tree Of Life V4 (http://itol.embl.de) [35]. Additionally, phylogenetic networks were conducted with the aim to detect recombination signatures in the analyzed population. These analyses were carried out in SplitsTree5 [36], using the neighbor-net method. We included as reference sequence the UA946 (LpanUA) sequence from Urrea et al. [27]. Additionally, we used as outgroup the *L. guyanensis* genome assembly from DDBJ/ENA/GenBank database (http://www.ebi.ac.uk/ena) under Run accession SRR8179913 (Lguy_SRR8179913).

### 2.7. Evaluation of Chromosome and Gene CNVs

For the chromosomal somy estimation, the median read depth of each chromosome was initially calculated (di). All positions with a read depth of >1 standard deviation away from this initial median were then removed, and the di was recalculated from high-quality reads. Subsequently, the median depth of the 35 chromosomes (dm) for *L. panamensis* was calculated, and the somy (S-value) of each chromosome was obtained with the following formula: S = 2 × di/dm [37]. The somy values calculated from sequencing data are averages across the potentially variable somy of these cells. For this reason, somy values may be non-integers, representing the mean value of a mixed population. The ranges of monosomy, disomy, trisomy, tetrasomy, and pentasomy were then used to define the full cell-normalized chromosome depth or somy (S) as S < 1.5, 1.5 ≤ S < 2.5, 2.5 ≤ S < 3.5, 3.5 ≤ S < 4.5, and 4.5 ≤ S < 5.5, respectively, as previously described [25]. As the depth of the genomes was sufficient, we did not investigate other normalization factors, such as various percentile depths or a statistically weighted normalization factor. To evaluate the CNVs at the gene level, we defined average haploid depth per gene without somy effect as d_HG_ and the full cell depth with somy effect as d_FG_. Their relationship was defined as *d_FG_* = *S* × *d_HG_*. We evaluated the gene or chromosome copy number by considering their biological and statistical significance. Significance was set at a z-score cutoff of >2 and adjusted *p*-value (Student’s *t*-test) of <0.05. Tandem gene arrays were defined as groups of homologous genes that were contiguously located on a chromosome. The heatmaps were created using the Heatmap3 package in R [38]. Finally, the genes that presented CNVs were subjected to Gene Ontology enrichment analyses using TriTrypDB tools (http://tritrypdb.org) with Fisher’s exact test used to maintain the FDR below 0.05. The GO terms were submitted to REVIGO [39].

### 2.8. SNP Estimations

To detect the SNPs, the reads were aligned to the reference UA946 *L. panamensis* genome sequence assembly using the SMALT program (version 0.7.4) (http://www.sanger.ac.uk/science/tools/smalt-0). Smalt options for exhaustive searching for optimal alignments and random mapping of multiple hit reads were used. The Picard program (version 1.85) (http://broadinstitute.github.io/picard/) was used for merging and sorting bam files and marking duplicated reads, as described previously [25]. The SNPs of <15 bp were identified using the population-based Unified Genotyper method in the Genome Analysis Toolkit (GATK) (version 3.4; https://software.broadinstitute.org/gatk/). Low-quality SNPs were filtered by GATK Variant Filtration criteria considered were QD < 2.0 || MQ < 40 || FS > 60.0 || ReadPosRankSum < −8.0. To avoid false negatives, the SNP quality cutoff was set at 300. All candidate SNPs were visually inspected in the Integrative Genomic Viewer (IGV_2_3_47) and SAMtools to avoid false positives. Once the SNPs were identified along the genomes, the allele frequency per chromosome was determined in each sample. Homozygous and heterozygous variant SNPs were determined from allele frequency estimation data, considering as homozygous variants to allele shift of < 0.2 or > 0.80 and as heterozygous variants to allele shifts between 0.2 and 0.8 [40]. Additionally, the SNPs shared between and within the three clades were included in a matrix Excel, which was used to perform the Venn diagram and to represent graphically the SNPs shared in a circular bar plot using an R script. 

The SnpEff program (version v4.1) [25] was used to classify all SNPs based on their functional impact. The SNPs were considered significantly different when the allele shift difference was at least 0.25 [40], with a Mann–Whitney U test *p*-value of <0.05. 

### 2.9. Data Availability

The dataset generated during the present study was deposited at DDBJ/ENA/GenBank under the accession number PRJEB35090.

## 3. Results

### 3.1. Identification of Leishmania Species

The clinical characteristics and the geographical distribution of the Colombian isolates included in the study are presented in Table 1 and Figure S1, respectively. Sanger sequencing analysis performed using as target the Cytb and HSP70 genes allowed us to identify that the samples analyzed corresponded to *L. panamensis* (Table S1). We also observed that all the samples were collected from the western part of Colombia: 63% of them obtained from Antioquia (12/19), 21% from Choco (4/19), and the remaining 16% from other areas (Santander, Bolivar, and Cauca). 

### 3.2. Nuclear and Mitochondrial Phylogenomic Inferences

**Figure 1:**
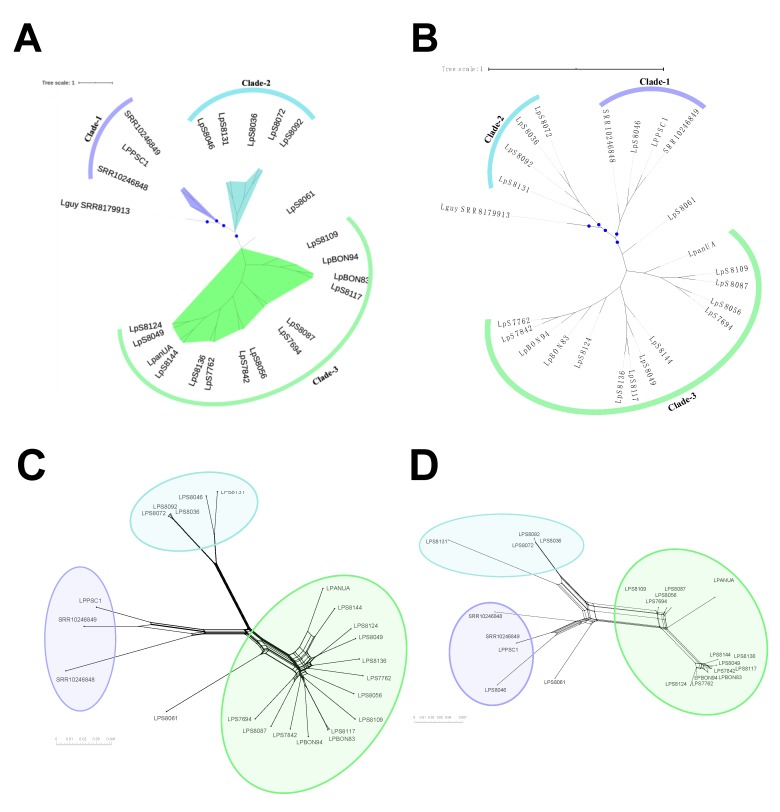
*Leishmania panamensis* phylogenomic relationships of nuclear and mitochondrial genomes. The trees located in the top of the figure represent the phylogenomic analysis based on nuclear (**A**) and mitochondrial: Maxicircle (**B**) single-nucleotide polymorphism (SNP) alignments of 22 genome sequences analyzed in this study, which were built under a Bayesian approach conducted in BEAST-2 program and using Lguy_SRR8179913 (*L. guyanensis*) as outgroup. LpanUA was used as a reference genome of *L. panamensis*. The posterior probabilities ≥ 0.8 are indicated by blue circles. The bottom panel represents the phylogenetic network (NeighborNet) constructed in SplitsTree 5, based on nuclear (**C**) and mitochondrial: Maxicircle (**D**) SNPs alignments for the 22 genomes analyzed.

The preliminary phylogenomic analyses based on nuclear (Figure S2A) and mitochondrial: Maxicircle (Figure S2B) genomes using the UA936 *L. panamensis* (LpanUA) genome sequence as reference and *L. guyanensis* (Lguy_SRR8179913) genome sequence as outgroup, allowed to confirm that all the genomes evaluated were closely related to LpanUA becoming additional evidence that these genomes belong to *L. panamensis.* The tree topology revealed the potential existence of three subpopulations clustered in well-supported nodes (with bootstrap ≥ 90.0) (Figure S2). The subsequent Bayesian phylogeographic analyses from nuclear and mitochondrial genomes (Figure 1A,B, respectively) showed consistently the existence of three main clusters, which were later confirmed by the topology of the phylogenetic networks using the network algorithm method (Figure 1C,D, respectively), supporting the hypothesis that *L. panamensis* is diversifying into three major clades having relationship with their geographical origin: Clade-1 (highlighted in purple) where all Panamanian sequences fell in (LPPSC-1, SRR10246848, SRR10246848), the Clade-2 (highlighted in sky blue), which included five Colombian genomes (LpS8036, LpS8046, LpS8172, LpS8192, and LpS8131) and the Clade-3 (highlighted in green), where the other Colombian genomes were clustered. Interestingly, the Clade-2, which included Colombian isolates collected from Chocó and Antioquia (two west Colombian regions located close to Panama), was closer to Clade-1 (which includes all isolates from Panama), than to the Clade-3, which included the other Colombian isolates collected inside the country (Antioquia, Cauca, and Bolivar). 

Another interesting result is associated with the LpS8061 sequence, the only genome that could not be assigned to any of the three major clades identified (Figure 1A–D), that was collected in Santander, a geographical region located in eastern Colombia. Finally, during the analysis of the mitochondrial genome (Figure 1B–D), two remarkable swapping events were identified in the phylogenetic tree (Figure 1B), that were supported by the phylogenetic network topology (Figure 1D). One of them involved the change of LpS8046 genome from Clade-1 to Clade-2 and the second event involved the identification of two possible sub-clades relatively distant in the Clade-3 (sub-clade-1 formed for LpS7762, LpS8124, LpBON94, LpS7842, LpBON83, LpS8117, LpS8136, Lp8049, and Lp8144 genomes, and sub-clade-2 for LpS7694, LpS8109, LpS8056, and LpS807 genomes); these possible clustering patterns were analyzed and compared with the clinical and demographic characteristics of each genome, however, in this study we did not observe any relationships among them. 

### 3.3. Evaluation of Chromosome and Gene CNVs

**Figure 2:**
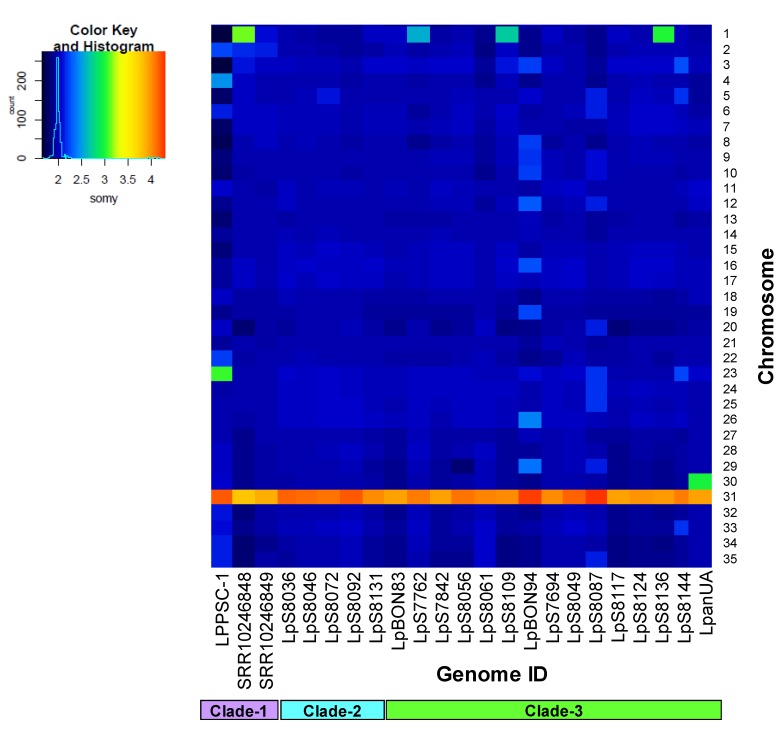
Dynamics of somy among the genomes of *Leishmania panamensis* analyzed. The heatmaps show median normalized read depths of 35 chromosomes (*y*-axis) identified in each genome of *L. panamensis* (*x*-axis) The color key indicates the somy value (S), which ranged from 1 to 5 as follows: monosomy, S < 1.5; disomy, 1.5 ≤ S < 2.5; trisomy, 2.5 ≤ S < 3.5; tetrasomy, 3.5 ≤ S < 4.5; and pentasomy, 4.5 ≤ S < 5.

We used the reads obtained in the sequencing to estimate the copy numbers per chromosome in all genomes analyzed. When we compared the somy values among them, we observed that in most of the genomes analyzed 34 of 35 chromosomes exhibited disomic behavior, except chromosome 31, which presented more than three copies in all genomes. Additionally, we observed that in some genomes of Clade-1 (SRR10246848) and of Clade-3 (LpS8109 and LpS8136), the chromosome 1 presented a trisomic behavior compared with the other genomes (Figure 2). The S-values were consistent with the somy values predicted based on the alternative allele frequency profiling. The allele frequency counts for each predicted heterozygous SNP did not exhibit discordance between read depths and allele frequencies, confirming the accuracy of the previously described somy profiles.

**Figure 3:**
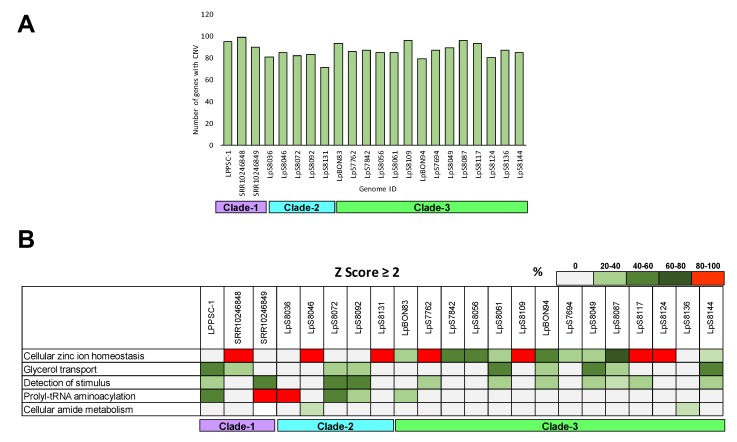
Evaluation of copy number variation (CNV) genes and ontology enrichment analyses. The figure shows the number of genes with CNVs in each genome analyzed (**A**) and the ontology enrichment analyses in the genes that present CNVs per sample (**B**). The color describes the percent of genes associated with each biological process per genome analyzed.

Subsequently, we evaluated the genes that presented CNVs (z-score cutoff >2 and adjusted *p*-value) and compared each genome included in the study; the results revealed minimal differences among them. The genomes that presented the lowest and highest numbers of genes with CNVs were LpS8131 and SRR10246848 (71 and 99 genes, respectively) (Figure 3A). A total of 44 genes presented CNVs in all the genome analyzed, seven of which (16%) were annotated as hypothetical proteins and the remaining 37 (84%) as having known functions. Their functional annotations included heat shock protein, α- and β-tubulin, amastin, histones, iron/zinc transporter, tuzin protein, and ATG8 (Table S2). Additionally, the Gene Ontology enrichment analysis of the genes that presented CNVs indicated that the enriched terms among them were mainly associated with cellular zinc ion homeostasis, glycerol transport, and stimulus detection. An interesting result was observed for the LpS8036 genome, with 100% of the genes presenting CNVs associated with prolyl-tRNA aminoacylation (Figure 3B). 

### 3.4. SNP Estimations

**Figure 4:**
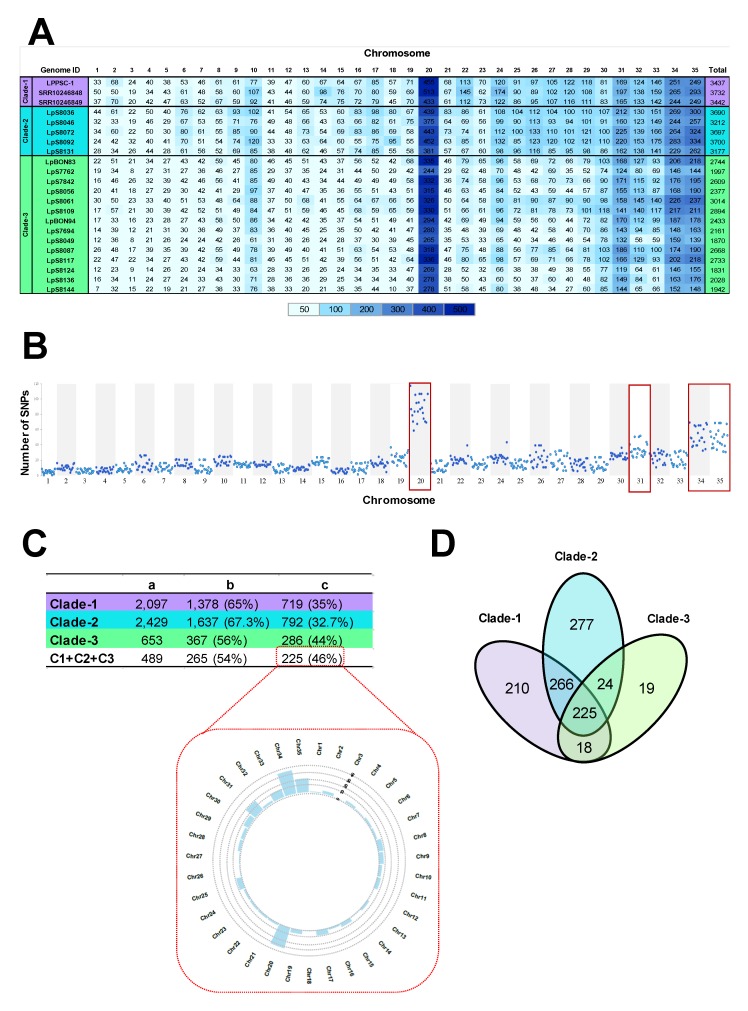
Overview of the SNPs identification results in genomes of *L. panamensis.* (**A**) Number of SNPs with functional impact per chromosome in each genome analyzed. The total number of SNPs in each genome is presented at the end of the table. (**B**) Density of SNPs per chromosome for every 10 kb of the genomes of *L. panamensis* herein analyzed. (**C**) Summary of the number of SNPs shared between and within each clade (C1+C2+C3 represents the number of SNPs shared between the three clades analyzed). (a) Total number of SNPs shared, (b) number of SNPs shared located in genes encoding hypothetical proteins, (c) number of SNPs shared located in genes encoding annotated proteins. The numbers in the parentheses represent the percent of SNPs. The bottom figure represents the number of SNPs shared per chromosome between three clades. (**D**) Total number of SNPs shared located in genes encoding known proteins, between and within three clades: Purple, sky blue, and green colors represent Clades 1, 2, and 3, respectively.

We analyzed and determined the number of SNPs in each of the genomes analyzed. The obtained results showed 61,388 SNPs in total. When we evaluated the potential effects of those SNPs on gene function, we identified SNPs with a moderate or high functional impact, approximately 2700 SNPs per genome, with the lowest (1831) and highest (3732) numbers of SNPs with functional impact identified in LpS8124 and SRR10246848 genomes, respectively (Figure 4A); considering the total number of variants, our results showed no relationship between the number of SNPs and the ploidy (e.g., chromosome 31: more than three copies); however, the number of SNPs was associated with the chromosome size because larger chromosomes presented a greater number of SNPs (chromosomes 20, 31, 34, and 35) than the shorter chromosomes (Figure 4A,B).

Of the SNPs with functional impact, we identified that 64.8% of them were homozygous SNPs and 35.2% heterozygous SNPs (Table S3), which describe the high genomic stability of *L. panamensis* (2700 SNPs per genome analyzed) compared with the stability of other *Leishmania* species (e.g., *L. braziliensis*, which presents an average of 17,000 SNPs per isolate). Most variants observed were synonymous, whereas the others affected the stop positions (Table 2). Several of SNPs identified are located in genes encoding hypothetical proteins, whereas the others are mainly located in genes encoding transporter proteins (pteridine transporter), surface proteins (amastin surface protein), and cytoskeletal proteins (β-tubulin). 

Later, we evaluated the SNPs shared between and within the clades identified. When we compared the genomes included on each clade, we observed that the Clade-3 had the lowest number of SNPs shared within the genomes (653 SNPs), compared with Clade-1 and Clade-2 where the number of SNPs shared was 2097 and 2429, respectively (Figure 4C); in general most of SNPs shared in each clade were located in genes encoding proteins associated with hypothetical function (56–67%) and the remaining located in genes with known function (33–46%). When we compared the three clades (Clade-1 + Clade-2 + Clade-3), we identified 489 SNPs shared between them (Figure 4C) of which 225 (46%) were mainly located in genes encoding transporter proteins (pteridine transporter, ABC transporter, folate/bioperin transporter, and iron/zinc transporter), intracellular degradation-associated proteins (ubiquitin-conjugating enzyme putative), host–pathogen interaction-associated proteins (amastin surface protein), or LPG biosynthesis-associated protein (galactofuranosyl glycosyltransferase) (Table S4). Additionally, the comparison across the three clades determined not only the presence of unique SNPs in each one of them, possibly associated with the geographical origin of the samples, but also the elevated number of SNPs shared between Clade-1 and -2 (266 SNPs) (Figure 4D).

**Figure 5:**
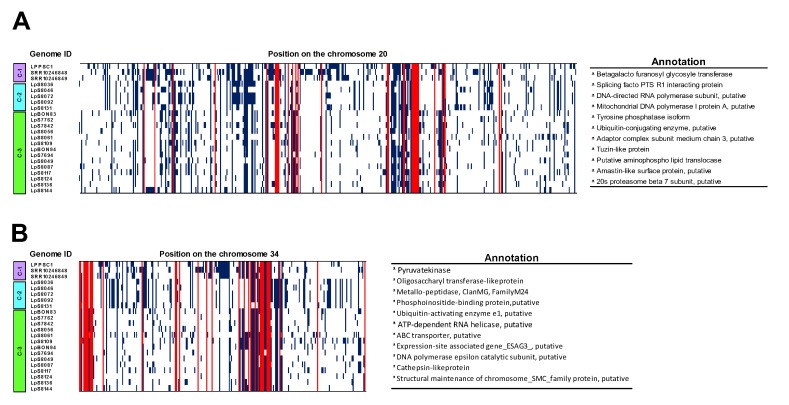
Overview of the SNPs identification results in chromosomes 20 and 34 of each genome analyzed. SNPs identified along chromosome 20 (**A**) and chromosome 34 (**B**). The dark blue line represents the presence of a SNP through each chromosome and the red line the location of shared SNPs among all genomes analyzed. The table on the right of each figure shows the annotation of the main genes with SNPs shared among three clades identified in this study.

Finally, and considering that chromosomes 20 and 34 were the chromosomes that presented the greatest number of SNPs shared between the three clades (31 and 34, respectively) (Figure 4C), we graphically presented the position of those SNPs across the chromosome, as well as the location and annotation (Figure 5A,B).

## 4. Discussion

Although the extensive literature has revealed intra- and interspecific genetic variability of some *Leishmania* species (mainly *L. donovani*, *L. major*, *L. tropica*) [23,30,41,42], little is known about the genetic variability of *Leishmania* (*Viannia*) species. The reported studies so far have focused on *L. braziliensis*, where the majority report consistent findings in describing its high intraspecific genetic diversity [16,26,43] even in cell populations isolated from a single *L. braziliensis* strain (“sub-strain”) [44], while in *L. panamensis* the results are not yet concordant, probably for the molecular approaches used. While studies using MLST reveal extreme genetic homogeneity within *L. panamensis* [10], others using MLEE [5], sequencing of specific genes (Cytb/HSP70) [6], AFLP [13], or microsatellites [14], have reported a high intraspecific genetic diversity in this species. Considering that DNA high-throughput sequencing has the potential to identify changes in the whole genome structure, we used this approach to reveal the intraspecific genomic variability of *L. panamensis* from Colombian clinical isolates and Panamanian genomes retrieved from databases.

Initially, and with the purpose of determining the relationship among the 22 genomes analyzed, we performed a Bayesian phylogenetic analysis on nuclear and mitochondrial genomes (Figure 1A–D). The results obtained both in nuclear and mitochondrial analysis, allowed us to identify the potential existence of three clades (Clade-1, Clade-2, and Clade-3). Clade-1 was formed by Panamanian sequences, Clade-2 was formed by genomic sequences from northwest Colombia (Chocó and Antioquia), and Clade-3 was formed by genomic sequences from other regions of Colombia (Bolivar, Cauca, and Antioquia). Interestingly the genomes that fell into the Clade-2 are closer to Clade-1 (Panamanian genomes) than Clade-3; those results could indicate that in Colombia two different phylogroups of *L. panamensis* can be found. Comparing the transmission cycles of *L. panamensis* in Colombia and Panama; the epidemiological landscape in Panama is mainly enzootic and involves different sand fly vectors (*Lutzomyia trapidoi* and *Lutzomyia panamensis*) [45,46] and vertebrate reservoirs (*Choloepus hoffmani* and *Bradypus griseus*) [46,47], while in Colombia, the transmission cycles are predominantly intra and peridomiciliary [5,48,49]. In addition, considering that some studies describe that the high transmission rate of *L. panamensis* appears to be mainly related to high population densities of animal reservoirs (*Choloepus hoffmani*) in areas of the old-growth forest [46,50]. We believe that the presence of the new clade (Clade-2) in the Colombian territory could be due to (i) the migration of these reservoirs from Panamá to Colombia as result of landscape alteration which triggers demographic changes that lead to occasional migrations out of the territory [46,51], (ii) occupational exposure to infectious sandfly bites when humans enter the forest [52,53], or (iii) human migration from or to Colombia [54,55].

Another possible explanation for the presence of two clades in Colombia, can be associated with possible disruptive segregation of ancestral populations of *L. panamensis,* similar to that proposed for *Triatoma dimidiata* in the transmission of *Trypanosoma cruzi* [54]. This hypothesis is supported by the wide variety of sandflies species that can transmit *Leishmania* in Colombia; and also, when we analyze the number of SNPs of Clade-3, this has the lowest number compared to other clades (653 SNPs) which could suggest it is an ancestral clade that has suffered a possible bottleneck. However, we believe that additional studies and a larger pool of Colombian and Panamanian genomes are necessary to confirm this hypothesis including Bayesian dating analysis that helps us to depict the emergence time scale of these clades. An additional observation about the genomic divergence found is associated with the geographical localization of the clades identified. While the genomes belonging to Clade-3 occupy Colombian mountain ecosystems (western cordillera), most of the genomes of the Clade-2 (specifically Chocó) and all genomes of Clade-1 are located outside of these mountainous regions, in dry and warm zones at lower altitudes (Figure S1), which suggest not only that the western cordillera is acting as geographic barrier to avoid the dispersion of native vectors species from the eastern to western region, but also, could be separating two divergent genetic clades producing a strong inter-population structure, as has been proposed for different Triatominae [53,54] and *Lutzomyia* [49,55,56] species. 

We identified a swapping event when we compared tree topologies of nuclear and mitochondrial genomes, where there is a marked change in clustering of a genome belonging to Clade-2 in the first case, to Clade-1 in the second, respectively (Figure 1). This finding may represent evidence of the existence of introgression events, the transfer of genetic material, usually via hybridization and backcrossing, from one entity (clade in this case) into the gene pool of a second divergent entity, which has been described as an alternative for the rapid evolution on ecological time scales playing a key role in response to environmental changes [57]. In addition, the evidence of two potential sub-clades detected in the mitochondrial genome phylogenies (Figure 1C,D) may suggest that genes carried in the mitochondrial genome are important for local adaptation and dispersion of *L. panamensis,* a validated theory for genes exposed to introgression in other species including *Trypanosoma cruzi* [57,58]. These phylogenetic signatures represent initial findings of the possible dispersal events of this new world *Leishmania* species; however, studies are required to clarify the population structure and the evolutionary forces that have shaped this divergence, through the development of studies with a larger number of samples, ideally involving other countries.

Subsequently, we analyzed the structural changes to chromosome level using the copy number variation as an indicator. Various studies have described that some *Leishmania* species (*L. infantum*, *L. major*, *L. donovani*, *L. amazonensis*, *L. braziliensis*, and *L. panamensis*) generate changes in the number of chromosomes as an environment-dependent mechanism, as well as an adaptation process in response to stress [24,25,28,37,59,60,61]. However, although this mechanism is shared between Old and New World *Leishmania* species, the changes in the number of chromosomes vary considerably across species. The results obtained in this study describe the homogeneity in somy across the *L. panamensis* genomes herein analyzed (Figure 2) and additionally confirm the high somy value in chromosome 31, which is characteristic of all the *Leishmania* species evaluated to date [20]. These results are consistent with the dynamics of ploidy observed in genomes SRR10246848 and SRR10246849 analyzed by Restrepo et al. [28]. The lack of genomic plasticity at the chromosomal level observed for *L. panamensis* has also been reported in other pathogens such as *T. brucei*; recently, a study has shown that none of the *T. brucei* subspecies present aneuploidy [62]. The low aneuploidy in these pathogens could be explained as a possible feature of adaptation of these parasites to human hosts or could be related to the loss of recombination events in this species, similar to that proposed for *T. brucei* [62]. This is also supported by the low number of SNPs found in the *L. panamensis* genomes analyzed which would explain a plausible mechanism of adaptation to human infection. This adaptation process has been clearly demonstrated in Colombian clinical isolates from an urban population survey conducted by our group where *L. panamensis* is the predominant species [5] compared with the enzootic population where *L. braziliensis* is the more prevalent species [6].

Another parameter evaluated in this study corresponded to local CNVs. The obtained results allowed us to identify a low number of genes with CNVs in the genomes analyzed (Figure 3A,B); these results reinforce the concept of a low level of structural variation in *L. panamensis*. Despite these findings, we analyzed the genes with CNVs and observed that 44 of them were shared among all the genomes. Interestingly, some of them have been previously identified by other authors and include genes involved in parasite survival and virulence [27,28], which indicate their importance during the adaptation to human hosts. Additionally, the ontology enrichment analysis revealed that most genes with CNVs were involved in cellular ion zinc homeostasis in many clinical isolates; some of these genes were previously identified in *L. infantum* [63], as reported by a previous study, wherein the authors have described the need of these genes in the parasite to maintain the intracellular concentrations of zinc and avoid the toxic effects of this metal. Considering the aforementioned findings, we believe that *L. panamensis*, similar to other *Leishmania* species, needs to maintain intracellular zinc homeostasis because of the importance of this metal as a cofactor of several hundred proteins involved in replication, infectivity, and virulence [64,65]. Another group of genes with CNVs observed in some genomes was associated with glycerol transport. Previous studies have described that glycerol can serve as a precursor for the synthesis of complex carbohydrates (such as β-mannan) or glycoconjugates (such as LPG) [66], suggesting that the transport of this component in *L. panamensis* is essential for its survival and infection establishment. In this context, natural selection could be occurring favoring that CNVs are located in pivotal genes for the survival of the parasites. Nevertheless, future studies should consider the effect of natural selection of microorganisms with low structural variation as *L. panamensis*.

The last genomic analysis used to evaluate the genetic variability among the genomes included in this study was associated with the identification of nucleotide-level variations (SNPs). Initially, we observed that the number of SNPs per genome in all the clades analyzed was low (≈1800 to ≈3700 SNPs) (Figure 4A), compared with the results obtained for other *Leishmania* (*Viannia*) species, such as *L. braziliensis*, where the number of variants observed in clinical isolates from Brazil ranged from ≈96,000 to ≈132,000 SNPs [26], in clinical isolates from Colombia was approximately 18,000 (data in preparation), and in *L. peruviana* isolates was approximately 27,000 [21]. These results together with the relationship between the number of homozygous/heterozygous SNP sites (39.785/21.603), observed in this study (Table S3), indicate not only a considerable genetic differentiation among species belonging to the same subgenus (*L. braziliensis*, *L. peruviana*, and *L. panamensis*) but also substantially lower variation in terms of SNPs in *L. panamensis*. We considered that the low genetic diversity observed in *L. panamensis*, might be shaped probably due to the process of adaptation to human infection. This could generate a bottleneck in this species that favored certain genotypes (founder effects) and promoted the stochastic loss of others, allowing the drift on its genomic diversity, as has previously been pointed out for *T. brucei* [67]. 

When we evaluated the SNPs shared within the genomes of each clade, we observed that despite geographic proximity into the genomes of Clade-2 and -3 (Figure S1), they presented a great difference regarding their genomic diversity. We observed a greater genetic homogeneity in the genomes belonging to the Clade-3 than in the genomes belonging to Clade-2 (Figure 4C). In addition to these findings, we observed that the genomes of Clade-2 present a similar number of SNPs (Figure 4A) and SNPs shared (Figure 4C), with the genomes belonging to the Clade-1 (Panamanian genomes). Interestingly, when we analyzed the relationship between the three clades, we observed that Clade-2 shared more SNPs with Clade-1 (266 SNPs) than with Clade-3 (24 SNPs) (Figure 4D). The genetic and geographic proximity of the genomes belonging to Clade-1 (2097 SNPs) and Clade-2 (2429 SNPs) and the difference regarding the genomes of Clade-3 (653 SNPs) suggest not only the presence of a new clade of *L. panamensis* in Colombia, but also the possible incorporation of it from Panama, probably due to migration of humans, vectors, or reservoirs towards our country. These results are very important since the incorporation of a new genotype could impact not only at the epidemiological level of CL but also probably affect the clinical and therapeutic response to antimonials. However, we believe that is necessary to analyze a larger number of Panamanian and Colombian genomes to confirm these hypotheses. Another interesting finding was associated with the 225 SNPs annotated, shared between the three clades analyzed (Figure 5A,B. Table S4), most of them previously described in the literature and involved in the different biological processes of the parasite [27,68]. 

## 5. Conclusions

In conclusion, this is the first study to report the intraspecific genomic variability of *L. panamensis*. The results demonstrate the existence of three well-supported clades as evidence of intraspecific divergence as well as the low structural variability in terms of somy, CNVs, and SNPs, which agree with a previous study [28]. Additionally, a particular phenomenon of adaptation to human infection as is the case of the Colombian clades due to their significant decrease in SNPs number was found. These findings suggest that this parasite does not require major structural changes to adapt to the vertebrate host and vectors responsible for the maintenance and transmission of CL but indeed drifts enough to maintain a stable and successful transmission cycle. However, we consider that additional analyses with a large number of genomes from different South American regions are necessary to confirm these findings. 

## Figures and Tables

**Table 1:** Clinical characteristics of each clinical isolate analyzed in the study.

Genome ID	Gender	Age (Years)	Origin	Number of Injuries	Lesion Type	Treatment
7694	Female	73	Antioquia	3	Ulcerative	Glucantime
7762	Male	14	Antioquia	1	Ulcerative	Glucantime
7842	Male	58	Antioquia	1	Ulcerative	Glucantime
8046	Male	21	Choco	3	Ulcerative	Miltefosine/Thermotherapy
8056	Male	21	Antioquia	3	Ulcerative	Miltefosine/Thermotherapy
8061	Female	70	Santander	3	Ulcerative	Glucantime
8072	Male	37	Antioquia	18	Ulcerative	ND
8092	Male	41	Antioquia	4	Nodular	Glucantime
8109	Male	33	Bolivar	6	Ulcerative	Glucantime
8036	Male	26	Choco	1	Scabs	ND
8049	Male	24	Antioquia	1	Ulcerative	Miltefosine/Thermotherapy
8087	Male	14	Antioquia	1	Ulcerative	Thermotherapy
8117	Male	29	Antioquia	1	Ulcerative	Miltefosine/Thermotherapy
8124	Male	22	Choco	3	Ulcerative and Nodular	Thermotherapy
8131	Male	32	Choco	1	Ulcerative	Miltefosine/Thermotherapy
8136	Female	31	Antioquia	1	Ulcerative	Glucantime
8144	Female	52	Antioquia	6	Ulcerative	Thermotherapy
BON 83	Male	25	Antioquia	2	Ulcerative	Glucantime
BON94	Male	19	Cauca	1	Ulcerative	Glucantime

ND. No Data.

**Table 2:** Selected set of SNPs with synonymous variant effect or relevant impact on gene function.

Genome ID	Number of Stop Lost	Number of Stop Gained	Synonymous Variant	Other Variants	Total
LPPSC-1	2	13	3399	23	3437
SRR10246848	2	12	3713	5	3732
SRR10246849	2	15	3415	10	3442
LpS8036	2	16	3668	4	3690
LpS8046	2	12	3193	5	3212
LpS8072	2	16	3675	4	3697
LpS8092	2	16	3679	3	3700
LpS8131	2	9	3161	5	3177
LpBON83	1	10	2727	6	2744
LpS7762	1	8	1986	2	1997
LpS7842	1	10	2594	4	2609
LpS8056	1	16	2354	6	2377
LpS8061	2	13	2994	5	3014
LpS8109	1	8	2878	7	2894
LpBON94	2	8	2419	4	2433
LpS7694	1	12	2145	3	2161
LpS8049	1	9	1854	6	1870
LpS8087	1	14	2648	5	2668
LpS8117	1	10	2716	6	2733
LpS8124	1	10	1814	6	1831
LpS8136	1	10	2010	7	2028
LpS8144	1	9	1926	6	1942

## Supplementary Materials

The following are available online at https://www.mdpi.com/2073-4425/11/3/252/s1, **Figure S1**: Geographical distribution of genomes included in the study. Georeferenced genomes discriminated by geographical localization. The maps were built on QGIS 3.6.0 based on the GPS coordinates of each isolation (QGIS Geographic Information System, Open Source Geospatial Foundation Project, http://qgis.osgeo.org). The circles represent each clade identified in this study. **Figure S2**: Preliminary Leishmania panamensis phylogenetic analysis of nuclear and mitochondrial genomes. The phylogenetic reconstructions represent the preliminary results that show the potential existence of three populations in the genome set of L. panamensis analyzed based on (A) nuclear and (B) mitochondrial (Maxicircle) SNPs alignments. LpanUA as reference genome of L. panamensis and Lguy_SRR8179913 (L. guyanensis) was used as an outgroup. The trees were built in FastTree double precision version 2.1.10 [31] and visualized in the interactive Tree Of Life V4 tool (http://itol.embl.de) [35], **Table S1**: Percent of similarity between the sequences of each Colombian clinical isolate with the sequences deposited on the Genebank database, in the two genes evaluated (Cytb and HSP70), **Table S2**: List of genes with copy number variations (z score cut off >2 and adjusted p-value) in all the genomes analyzed, **Table S3**: Total number of homozygous and heterozygous SNPs found in the genomes analyzed. **Table S4**: SNPs shared among the three clades identified.
