# Supplementary material for: Intraspecific Genomic Divergence and Minor Structural Variations in Leishmania (Viannia) panamensis
Source: Genes (Basel). 2020 Feb 27;11(3):252. doi: 10.3390/genes11030252 (PMC7140786; doi:10.3390/genes11030252)
Supplement: Supplementary file 1 [file genes-11-00252-s001.pdf]

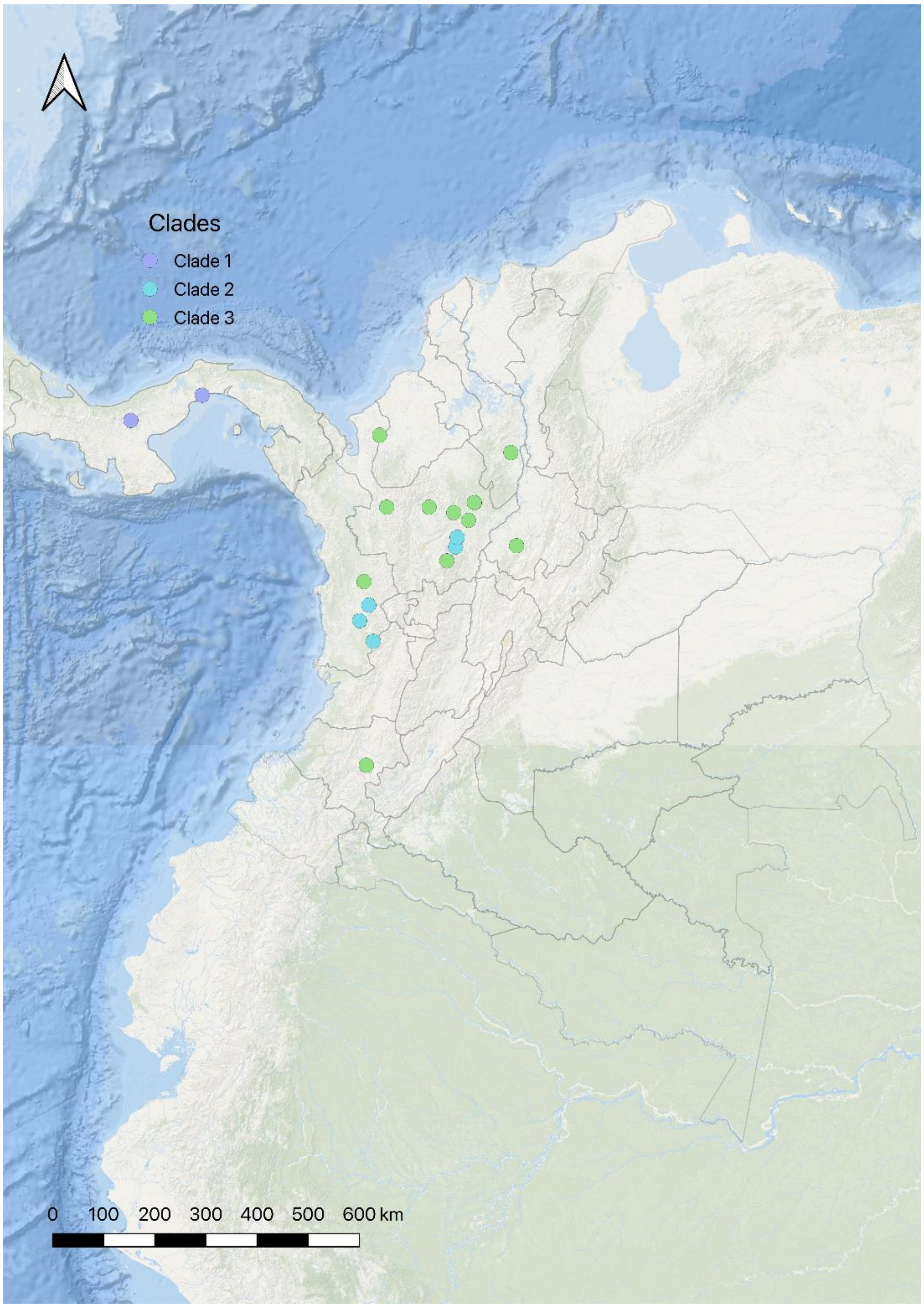

Figure S1: Geographical distribution of genomes included in the study. Georeferenced genomes discriminated by geographical localization. The maps were built on QGIS 3.6.0 based on the GPS coordinates of each isolation (QGIS Geographic Information System, Open Source Geospatial Foundation Project, http://qgis.osgeo.org). The circles represent each clade identified in this study.

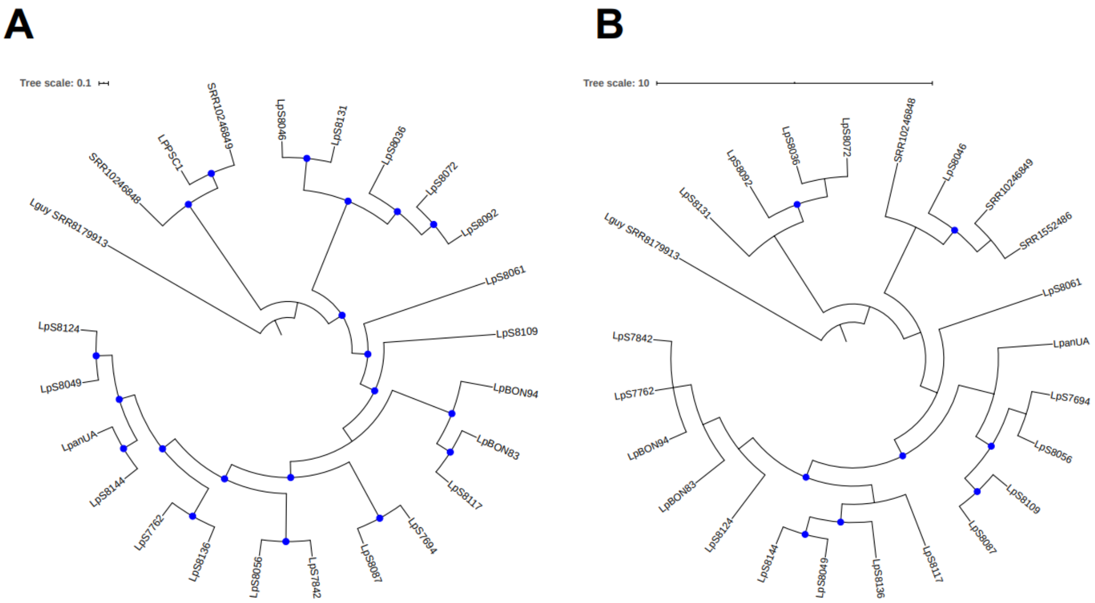

Figure S2: Preliminary Leishmania panamensis phylogenetic analysis of nuclear and mitochondrial genomes. The phylogenetic reconstructions represent the preliminary results that show the potential existence of three populations in the genome set of L. panamensis analyzed based on (A) nuclear and (B) mitochondrial (Maxicircle) SNPs alignments. LpanUA as reference genome of L. panamensis and Lguy_SRR8179913 (L. guyanensis) was used as an outgroup. The trees were built in FastTree double precision version 2.1.10 [31] and visualized in the interactive Tree Of Life V4 tool (http://itol.embl.de) [35],

Table S1: Percent of similarity between the sequences of each Colombian clinical isolate with the sequences deposited on the Genebank database, in the two genes evaluated (Cytb and HSP70)

Genome ID	Cytb gene		HSP70 gene	
	E value	Per. Ident	E value	Per. Ident
LpBON83	0	99.82%	2.00E-144	98.91%
LpBON94	0	99.65%	3.00E-147	98.99%
LpS7694	0	99.16%	7.00E-138	98.93%
LpS7762	0	99.31%	7.00E-148	99.00%
LpS7842	0	99.46%	2.00E-147	99.33%
LpS8036	0	99.48%	3.00E-146	98.93%
LpS8046	0	99.45%	5.00E-149	98.97%
LpS8049	0	99.48%	1.00E-150	98.99%
LpS8056	0	99.47%	2.00E-149	99.08%
LpS8061	0	98.79%	6.00E-144	98.33%
LpS8072	0	99.46%	3.00E-141	98.63%
LpS8087	0	99.65%	5.00E-149	99.00%
LpS8092	0	99.12%	1.00E-150	98.70%
LpS8109	0	99.65%	3.00E-147	98.68%
LpS8117	0	98.94%	1.00E-150	98.70%
LpS8124	0	98.80%	4.00E-150	98.69%
LpS8131	0	99.40%	7.00E-148	98.68%
LpS8136	0	98.90%	3.00E-152	98.71%
LpS8144	0	99.70%	9.00E-152	99.34%

Table S2: List of genes with copy number variations (z score cut off >2 and adjusted p-value) in all the genomes analyzed

Gene id	Chromosome	Start	End	Gene annotation	LPPSC-1	SRR10246848	SRR10246849	LpS8036	LpS8046	LpS8072	LpS8092	LpS8131	LpBON83	LpS7762	LpS7842	LpS8056	LpS8061	LpS8109	LpBON94	LpS7694	LpS8049	LpS8087	LpS8117	LpS8124	LpS8136	LpS8144
LpanUA.04.0250	LpanUA_04_V6	48630	50375	hypothetical_protein,_c	14.445	14.014	18.309	13.320	8.068	13.233	13.320	13.730	15.945	14.844	14.745	11.677	15.813	14.022	11.089	20.414	12.775	21.659	15.560	13.746	16.516	15.604
LpanUA.05.0520	LpanUA_05_V6	168946	170670	ATPase_alpha_subunit	5.260	5.131	3.240	2.789	2.836	2.623	2.730	2.512	3.276	2.770	2.798	3.274	2.861	2.907	2.788	2.977	3.081	2.772	3.185	2.928	2.881	3.081
LpanUA.08.0670	LpanUA_00_V6	18271	18858	amastin-like_protein	6.259	6.108	8.081	3.196	4.285	3.307	3.730	5.030	5.377	4.730	6.498	8.588	7.932	7.684	5.134	5.450	6.762	6.798	5.376	7.200	6.973	5.755
LpanUA.08.0820	LpanUA_08_V6	372337	373662	cathepsin_L-like_protea	2.590	3.304	3.012	2.549	2.463	2.675	2.465	2.288	3.189	2.738	3.158	2.742	2.635	2.917	2.125	3.183	2.814	3.304	3.141	3.095	2.952	2.970
LpanUA.08.0990	LpanUA_08_V6	278276	278839	amastin-like_protein	36.277	19.041	10.623	14.406	9.178	14.573	14.574	8.616	12.643	9.688	12.416	10.349	19.238	14.641	9.278	10.134	5.549	12.912	12.036	5.547	10.024	8.768
LpanUA.08.1025	LpanUA_08_V6	276815	277417	tuzin-like,_putative	15.818	17.214	10.910	7.863	5.423	8.892	8.278	5.386	6.553	4.990	6.360	5.773	12.108	8.766	4.997	5.635	3.023	6.978	7.778	3.206	6.315	5.212
LpanUA.09.0180	LpanUA_09_V6	63619	63993	ATG8/AUT7/APG8/PAZ2,	22.516	15.856	19.936	13.050	8.515	13.052	12.569	15.351	15.601	11.381	17.826	20.100	15.872	19.949	15.411	17.879	15.582	16.020	16.160	17.500	11.653	16.075
LpanUA.09.1075	LpanUA_09_V6	414178	414648	term=tRNA-Arg 15.009 2	14.768	25.210	15.116	12.799	9.834	12.962	13.215	12.122	20.996	15.981	17.322	19.958	19.820	17.512	20.076	18.450	21.876	18.798	22.376	20.574	19.656	17.815
LpanUA.10.0510.N	LpanUA_10_V6	195968	197454	metallo-peptidase,_Clar	10.740	10.491	4.974	5.974	11.784	6.337	6.276	6.362	10.454	4.613	3.866	3.514	16.754	17.292	4.975	2.054	5.237	3.393	10.660	5.574	5.583	2.931
LpanUA.10.0540.N	LpanUA_10_V6	214644	215936	metallo-peptidase,_Clar	8.087	7.895	6.387	2.732	2.018	2.806	2.634	2.182	5.475	4.602	6.761	5.597	5.500	4.008	5.361	6.553	2.856	4.378	5.540	3.159	6.589	2.853
LpanUA.11.1170	LpanUA_06_V6	509692	510681	TATE_DNA_transposons	2.661	2.588	4.188	4.992	5.000	4.657	5.278	5.187	5.024	3.217	3.650	6.795	6.560	4.051	5.169	4.547	4.803	6.805	5.951	3.605	3.441	3.864
LpanUA.13.0190	LpanUA_13_V6	97490	98845	alpha_tubulin	18.706	14.059	23.395	30.357	35.144	29.560	16.383	12.052	20.195	25.349	34.128	24.149	17.352	25.710	35.171	31.109	12.620	26.153	14.946	13.300	26.622	28.498
LpanUA.13.2690	LpanUA_13_V6	66172	68502	hypothetical_protein,_c	2.506	4.728	4.606	3.915	4.784	3.566	3.441	3.514	4.643	4.065	4.590	4.183	2.433	4.330	3.530	4.667	3.912	5.362	4.873	4.246	4.437	3.554
LpanUA.16.0605	LpanUA_16_V6	214686	214940	histone_H3-like,_putativ	32.726	58.567	60.136	7.714	8.209	7.133	6.228	12.787	8.428	10.058	7.861	8.679	7.751	12.691	6.155	9.502	14.519	7.779	15.414	16.987	12.918	13.075
LpanUA.16.1480	LpanUA_16_V6	577144	578928	paraflagellar_rod_protei	5.282	2.609	3.773	3.120	2.268	2.940	2.612	3.383	4.104	4.495	3.315	2.802	2.332	3.921	3.254	3.785	4.008	3.027	4.458	4.093	2.594	2.611
LpanUA.17.0005	LpanUA_17_V6	32	1132	TATE_DNA_transposons	7.722	7.539	5.031	2.509	6.318	2.698	2.566	5.058	11.179	9.376	10.348	11.398	8.545	7.770	11.131	10.851	11.997	12.099	11.719	9.976	10.262	12.538
LpanUA.17.0090	LpanUA_17_V6	27886	29235	elongation_factor_1-alp	31.278	30.579	29.864	23.388	18.256	23.428	23.940	21.959	26.318	25.245	27.388	22.120	23.843	26.982	21.611	22.210	20.596	22.082	26.298	19.718	22.517	20.984
LpanUA.19.1055	LpanUA_19_V6	410950	411507	NI	8.990	8.779	11.942	6.652	7.269	6.637	7.142	5.993	7.139	6.504	7.964	9.856	8.866	9.791	7.307	6.398	8.077	9.093	6.774	9.363	8.239	8.590
LpanUA.19.1905	LpanUA_19_V6	662940	663185	hypothetical_protein,_u	10.094	9.859	16.617	11.207	7.281	11.928	11.326	11.050	12.440	15.413	14.241	14.836	15.456	14.875	10.865	13.144	13.826	14.161	13.022	12.664	11.976	16.866
LpanUA.20.0483.N	LpanUA_20_V6	255787	256326	amastin	11.812	11.540	11.426	3.388	3.317	2.780	2.483	4.180	3.401	2.982	2.322	3.229	3.415	3.593	2.842	3.058	3.588	3.666	4.891	4.715	4.627	4.614
LpanUA.20.0484	LpanUA_20_V6	256444	257028	tuzin-like,_putative	19.742	19.296	18.822	12.764	23.270	12.775	12.342	25.896	15.299	13.138	10.474	16.187	15.322	15.439	14.649	14.116	12.380	14.305	17.922	16.665	16.144	16.719
LpanUA.20.0946	LpanUA_20_V6	450882	452090	tuzin-like,_putative	3.302	3.095	3.313	2.383	2.463	2.323	2.553	2.431	2.427	2.121	2.589	2.471	2.508	2.776	2.566	2.672	2.602	2.603	3.058	2.779	2.632	2.879
LpanUA.20.1045	LpanUA_20_V6	511604	512074	tuzin-like,_putative	9.318	9.100	8.716	4.615	4.975	3.991	4.468	7.570	6.952	3.999	4.820	4.922	5.854	5.959	7.215	7.581	4.208	6.312	7.162	4.628	5.923	6.269
LpanUA.20.1257.N	LpanUA_20_V6	817516	818409		19.875	31.201	42.641	22.023	22.102	21.127	21.649	25.869	21.683	21.318	13.146	21.791	16.771	22.172	17.170	16.657	28.823	20.020	26.034	25.661	23.411	22.178
LpanUA.20.1444	LpanUA_20_V6	743109	743609	ubiquitin-conjugating_e	8.019	7.829	8.097	7.001	6.394	6.989	7.112	9.159	9.891	8.266	8.055	8.043	8.779	9.467	9.500	11.880	8.957	10.217	10.138	8.631	9.563	10.513
LpanUA.20.2375	LpanUA_20_V6	1217866	1218792	tuzin-like_protein	9.659	9.434	17.087	5.574	10.098	5.542	5.631	5.930	9.953	9.094	9.111	9.826	8.563	12.354	8.029	7.502	9.463	8.762	10.435	7.538	9.315	10.033
LpanUA.20.2376	LpanUA_20_V6	1219011	1219502	tuzin-like,_putative	8.040	7.849	15.990	2.523	6.444	2.545	2.188	2.324	6.053	6.870	6.692	7.018	6.192	9.367	4.129	4.468	7.336	4.422	7.006	5.632	5.807	6.537
LpanUA.20.2870	LpanUA_20_V6	1413507	1414118	amastin-like_protein	12.623	12.332	5.844	5.457	6.381	5.412	5.373	5.135	7.089	6.949	9.658	5.576	6.395	6.156	6.730	7.356	8.688	9.347	7.444	7.655	6.520	11.780
LpanUA.20.4300	LpanUA_20_V6	1404887	1405465	amastin-like_surface_pr	9.681	9.455	12.243	3.832	3.404	3.640	3.434	4.530	3.570	3.741	2.829	3.867	3.456	3.944	3.157	3.004	5.294	3.575	4.484	4.249	4.205	4.094
LpanUA.21.0010	LpanUA_21_V6	12	1718	pyruvate_kinase	2.506	2.437	2.927	3.112	2.614	3.295	3.328	3.580	2.518	2.314	2.532	2.741	3.586	2.267	2.956	2.807	3.497	3.962	2.771	2.133	2.298	3.255
LpanUA.27.2810	LpanUA_05_V6	476185	477094	hypothetical_protein	4.509	4.396	8.543	6.836	3.495	5.920	6.155	6.849	6.407	9.663	7.422	9.248	9.844	8.289	6.525	6.499	8.717	7.055	7.193	7.330	5.306	9.589
LpanUA.27.2840	LpanUA_05_V6	473685	475874	metallo-peptidase,_Clar	4.741	4.623	8.944	8.546	5.746	8.191	7.874	8.879	6.771	9.518	8.235	9.077	11.183	9.147	7.114	7.487	8.280	7.400	6.930	7.591	6.253	10.025
LpanUA.28.2110	LpanUA_28_V6	748347	749402	zinc_transporter,_putati	5.967	5.822	7.061	2.754	5.477	2.866	2.869	4.230	5.706	4.262	4.848	4.548	4.120	4.702	4.348	4.937	4.693	4.467	6.021	4.732	4.744	3.285
LpanUA.28.2990	LpanUA_28_V6	1075126	1077090	heat-shock_protein_hsp	6.110	5.962	4.948	4.591	7.160	4.478	4.302	4.643	5.915	3.542	4.047	5.613	3.781	6.276	4.283	5.512	5.080	4.799	6.658	5.560	5.443	4.767
LpanUA.30.1520	LpanUA_30_V6	469311	469769	ama1_protein,_putative	8.540	8.339	8.157	5.491	5.276	5.516	5.444	5.109	5.931	5.761	5.555	4.991	6.293	5.468	5.192	5.536	6.330	5.947	6.204	10.992	4.866	5.221
LpanUA.32.3900	LpanUA_32_V6	1418541	1419629	3-hydroxyisobutyryl-coe	5.282	2.327	3.014	3.364	3.254	3.295	3.023	2.996	4.879	3.263	5.472	4.284	2.587	4.115	2.763	3.557	4.458	4.195	5.038	5.978	5.211	5.088
LpanUA.33.0340	LpanUA_33_V6	128882	130996	heat_shock_protein_83-	12.965	12.667	18.065	13.823	18.045	13.859	13.875	15.163	18.516	15.358	19.203	15.849	15.181	14.920	15.899	17.647	17.843	18.346	19.109	22.461	17.397	14.686
LpanUA.33.0960	LpanUA_33_V6	301688	303019	beta-tubulin	32.241	35.587	42.701	37.839	32.731	37.647	37.742	29.319	34.583	32.802	36.926	35.723	38.372	33.264	38.765	36.619	37.807	33.253	36.240	33.946	36.197	38.438
LpanUA.33.1880	LpanUA_33_V6	669291	670709	peptidase_M20/M25/M4	10.704	13.476	4.330	26.803	20.606	25.942	25.883	28.293	8.110	12.018	11.701	12.731	15.288	15.536	18.000	12.811	8.537	10.691	8.041	12.461	11.861	10.707
LpanUA.34.0060	LpanUA_34_V6	2308	3582	pyruvate_kinase	13.902	13.583	5.693	6.639	5.163	7.236	7.078	5.571	4.240	4.275	3.935	4.818	4.618	5.622	4.636	3.532	4.558	4.845	4.174	3.739	4.691	4.587
LpanUA.34.1800	LpanUA_34_V6	688960	689877	60S_ribosomal_protein_	2.083	3.679	3.447	2.640	2.036	3.001	2.834	2.540	2.686	4.990	2.084	2.663	5.087	2.466	2.076	2.819	2.255	2.966	2.577	2.735	2.867	3.093
LpanUA.34.ncRNA	LpanUA_05_V6	466082	467038	NI	8.996	10.552	21.098	5.377	7.462	5.235	5.373	5.833	9.149	8.692	8.425	5.761	5.248	7.040	5.689	6.314	8.547	5.705	10.104	9.032	9.445	10.202
LpanUA.35.0270	LpanUA_35_V6	45564	48101	elongation_factor_2	7.853	6.994	5.775	4.826	4.159	4.356	4.539	4.565	5.763	4.298	6.870	5.048	4.806	6.156	4.184	5.404	4.558	5.139	5.663	4.962	4.888	4.974
LpanUA.35.3755	LpanUA_35_V6	1329857	1330099	putative_polyubiquitin	59.893	121.897	124.860	12.600	7.448	13.830	12.529	10.124	12.360	12.478	17.866	15.932	10.349	11.453	7.978	13.120	5.771	9.005	12.888	10.144	13.945	12.942

NI: No information

Table S3: Total number of homozygous and heterozygous SNPs found in the genomes analyzed.

Genome ID	Homo	Hetero	Total
LPPSC-1	2.774	649	3.423
SRR10246848	3.153	579	3.732
SRR10246849	2.896	546	3.442
LpS8036	2.271	1.419	3.690
LpS8046	2.694	518	3.212
LpS8072	2.289	1.408	3.697
LpS8092	2.289	1.411	3.700
LpS8131	2.761	416	3.177
LpBON83	1.165	1.579	2.744
LpS7762	1.508	489	1.997
LpS7842	1138	1471	2.609
LpS8056	1.355	1.022	2.377
LpS8061	2.456	558	3.014
LpS8109	1.344	1.550	2.894
LpBON94	1.465	968	2.433
LpS7694	1.634	527	2.161
LpS8049	1.214	656	1.870
LpS8087	1110	1558	2.668
LpS8117	1139	1594	2.733
LpS8124	1.260	571	1.831
LpS8136	1.335	693	2.028
LpS8144	521	1421	1.942

Table S4: SNPs shared among the three clades identified.

a: SNP position on the gene

b: Change of nucleotide respect to reference (Ref/Alt)

Gene id	a	b	Funtional Impact	Gene Annotation
LpanUA.01.0080	16203	T/C	MODERATE	carboxylase,putative
LpanUA.02.0120	25230	G/A	MODERATE	exportinT tRNAexportin -likeprotein
LpanUA.02.0160	96145	C/T	MODERATE	phosphoglycanbeta1,3galactosyltransferase
LpanUA.02.0550	246457	G/C	MODERATE	RetrotransposableelementSLACS
LpanUA.02.0550	247375	A/G	MODERATE	RetrotransposableelementSLACS
LpanUA.04.0060	10361	A/G	MODERATE	pteridinetransporter truncated ,putative
LpanUA.04.0170	44238	A/G	MODERATE	exosomecomplexexonucleaseRRP40,putative
LpanUA.04.0810	300310	T/C	MODERATE	serinepeptidase,ClanS-,familyS54,putative
LpanUA.05.0540	176493	T/C	MODERATE	kinetoplast-associatedprotein-likeprotein
LpanUA.06.0080	38590	T/C	MODERATE	ABCtransporter,putative
LpanUA.06.0080	38591	A/G	MODERATE	ABCtransporter,putative
LpanUA.06.0760	307077	A/T	MODERATE	RNA-bindingprotein-likeprotein
LpanUA.07.1250	563972	C/T	HIGH	phosphoglycanbeta1,3galactosyltransferase1
LpanUA.07.1250	564585	A/G	MODERATE	phosphoglycanbeta1,3galactosyltransferase1
LpanUA.07.1250	564677	A/G	MODERATE	phosphoglycanbeta1,3galactosyltransferase1
LpanUA.08.0637	232774	C/T	MODERATE	amastin-likeprotein
LpanUA.08.0637	233016	A/C	MODERATE	amastin-likeprotein
LpanUA.08.1025	276898	G/A	HIGH	tuzin-like,putative
LpanUA.10.1525	292330	G/A	MODERATE	metallo-peptidase,ClanMA M ,FamilyM8
LpanUA.10.1525	292348	A/G	MODERATE	metallo-peptidase,ClanMA M ,FamilyM8
LpanUA.10.1525	292350	A/T	MODERATE	metallo-peptidase,ClanMA M ,FamilyM8
LpanUA.10.1525	292380	C/T	MODERATE	metallo-peptidase,ClanMA M ,FamilyM8

LpanUA.10.1525	292393	T/C	MODERATE	metallo-peptidase,ClanMA M ,FamilyM8
LpanUA.09.0004	418	A/T	MODERATE	HypotheticalTATEDNATransposon fragment
LpanUA.09.0004	447	T/C	MODERATE	HypotheticalTATEDNATransposon fragment
LpanUA.09.0180	63974	G/C	MODERATE	ATG8/AUT7/APG8/PAZ2,putative
LpanUA.09.0180	63988	G/A	MODERATE	ATG8/AUT7/APG8/PAZ2,putative
LpanUA.09.0880	323392	A/C	MODERATE	kinetoplast-associatedprotein-likeprotein
LpanUA.09.1400	530588	T/C	MODERATE	histoneH2B
LpanUA.09.1400	530630	T/C	MODERATE	histoneH2B
LpanUA.10.0370	138177	C/G	MODERATE	folate/biopterintransporter,putative
LpanUA.10.0370	138646	C/T	MODERATE	folate/biopterintransporter,putative
LpanUA.10.0470	191189	A/G	MODERATE	metallo-peptidase,ClanMA M ,FamilyM8
LpanUA.10.0520	199389	C/G	MODERATE	metallo-peptidase,ClanMA M ,FamilyM8
LpanUA.10.1305	488351	T/C	MODERATE	Putativeflagellarprotofilamentribbonprotein-likeprotein
LpanUA.11.0950	496915	C/G	MODERATE	CyclinA/CDK2-associatedprotein
LpanUA.11.0960	502662	C/T	MODERATE	ABCtransporter,putative
LpanUA.11.0960	502834	T/C	MODERATE	ABCtransporter,putative
LpanUA.11.0960	505710	T/C	MODERATE	ABCtransporter,putative
LpanUA.11.0960	506019	A/G	MODERATE	ABCtransporter,putative
LpanUA.11.1020	538521	G/A	MODERATE	ABCtransporter,putative
LpanUA.13.0120	43785	C/G	MODERATE	MCAK-likekinesin,putative
LpanUA.13.1380	529802	C/T	MODERATE	phospholipid-transportingATPase1-likeprotein
LpanUA.13.1380	531168	T/C	MODERATE	phospholipid-transportingATPase1-likeprotein
LpanUA.13.1380	531258	G/T	MODERATE	phospholipid-transportingATPase1-likeprotein
LpanUA.14.1150	524829	T/C	MODERATE	tubfamilyprotein-likeprotein
LpanUA.15.0005	1477	A/G	MODERATE	phosphoglycanbeta1,3galactosyltransferase1
LpanUA.15.0005	1830	A/G	MODERATE	phosphoglycanbeta1,3galactosyltransferase1
LpanUA.15.0005	2244	C/T	MODERATE	phosphoglycanbeta1,3galactosyltransferase1
LpanUA.15.0005	2524	T/C	MODERATE	phosphoglycanbeta1,3galactosyltransferase1
LpanUA.15.0005	2599	C/T	MODERATE	phosphoglycanbeta1,3galactosyltransferase1
LpanUA.15.0005	2640	A/G	HIGH	phosphoglycanbeta1,3galactosyltransferase1
LpanUA.16.0500	162107	T/C	MODERATE	fucosekinase,putative
LpanUA.16.0500	162830	A/G	MODERATE	fucosekinase,putative
LpanUA.16.1600	632130	A/G	MODERATE	DNApolymeraseIalphacatalyticsubunit,putative

LpanUA.17.0100	42474	C/T	MODERATE	receptor-typeadenylatecyclase,putative
LpanUA.17.0100	42566	A/G	MODERATE	receptor-typeadenylatecyclase,putative
LpanUA.17.0100	42626	T/C	MODERATE	receptor-typeadenylatecyclase,putative
LpanUA.17.0100	42659	T/C	MODERATE	receptor-typeadenylatecyclase,putative
LpanUA.17.0100	42686	G/A	MODERATE	receptor-typeadenylatecyclase,putative
LpanUA.17.0100	43863	A/G	MODERATE	receptor-typeadenylatecyclase,putative
LpanUA.17.0100	43887	C/T	MODERATE	receptor-typeadenylatecyclase,putative
LpanUA.17.0100	44629	C/A	MODERATE	receptor-typeadenylatecyclase,putative
LpanUA.18.1010	386778	A/G	MODERATE	mannosyltransferase-II,putative
LpanUA.19.1010	303832	T/A	MODERATE	kinesin,putative
LpanUA.08.1050	408666	G/T	MODERATE	amastin-likeprotein
LpanUA.08.1050	408767	T/A	MODERATE	amastin-likeprotein
LpanUA.08.1050	408774	A/G	MODERATE	amastin-likeprotein
LpanUA.19.1560	560130	G/A	MODERATE	glyceroluptakeprotein,putative
LpanUA.19.1740	611305	C/T	MODERATE	proteinkinase,putative
LpanUA.20.0480	245154	G/A	MODERATE	betagalactofuranosylglycosyletransferase
LpanUA.20.0482	252171	A/G	MODERATE	splicingfactorPTSR1interactingprotein
LpanUA.20.0485	259990	G/C	MODERATE	amastin
LpanUA.20.0860	430357	T/A	MODERATE	DNA-directedRNApolymerasesubunit,putative
LpanUA.20.1045	511780	T/A	MODERATE	tuzin-like,putative
LpanUA.20.1046	512740	G/A	MODERATE	tuzin-likeprotein
LpanUA.20.1046	512823	C/G	MODERATE	tuzin-likeprotein
LpanUA.20.1046	512830	A/G	MODERATE	tuzin-likeprotein
LpanUA.20.1046	512851	A/G	MODERATE	tuzin-likeprotein
LpanUA.20.1290	607199	T/C	MODERATE	mitochondrialDNApolymeraseIproteinA,putative
LpanUA.20.1433	688751	A/G	MODERATE	tyrosinephosphataseisoform
LpanUA.20.1444	743134	A/G	MODERATE	ubiquitin-conjugatingenzyme,putative
LpanUA.20.1444	743303	G/A	MODERATE	ubiquitin-conjugatingenzyme,putative
LpanUA.20.1256	809270	C/G	MODERATE	amastin-likeprotein
LpanUA.20.2150	1134112	T/C	MODERATE	adaptorcomplexsubunitmediumchain3,putative
LpanUA.20.2370	1216110	T/C	MODERATE	amastin-likesurfaceprotein,putative
LpanUA.20.2370	1216121	A/C	MODERATE	amastin-likesurfaceprotein,putative
LpanUA.20.2375	1218521	A/G	MODERATE	tuzin-likeprotein

LpanUA.20.2375	1218543	T/A	MODERATE	tuzin-likeprotein
LpanUA.20.2376	1219299	T/A	MODERATE	tuzin-like,putative
LpanUA.20.2376	1219306	A/G	MODERATE	tuzin-like,putative
LpanUA.20.2376	1219428	G/A	MODERATE	tuzin-like,putative
LpanUA.20.2376	1219434	C/A	MODERATE	tuzin-like,putative
LpanUA.20.2376	1219451	C/G	MODERATE	tuzin-like,putative
LpanUA.20.2376	1219452	G/C	MODERATE	tuzin-like,putative
LpanUA.20.2376	1219498	A/G	MODERATE	tuzin-like,putative
LpanUA.20.2800	1355587	C/G	MODERATE	putativeaminophospholipidtranslocase
LpanUA.20.0950	1397977	G/A	MODERATE	amastin-likesurfaceprotein,putative
LpanUA.20.4300	1405183	G/C	MODERATE	amastin-likesurfaceprotein,putative
LpanUA.20.3950	1767581	T/C	MODERATE	20sproteasomebeta7subunit, putative
LpanUA.20.3950	1767781	T/C	MODERATE	20sproteasomebeta7subunit, putative
LpanUA.21.0010	170	C/T	MODERATE	pyruvatekinase
LpanUA.21.0010	1063	A/C	MODERATE	pyruvatekinase
LpanUA.21.0010	1148	A/G	MODERATE	pyruvatekinase
LpanUA.22.0090	31318	A/G	MODERATE	mRNAcapmethyltransferase-likeprotein
LpanUA.22.0630	261647	T/G	MODERATE	5'a2rel-relatedprotein
LpanUA.22.1440	602724	A/G	MODERATE	alanyl-tRNAsynthetase,putative
LpanUA.23.0040	10655	A/C	MODERATE	betapropellerprotein,putative
LpanUA.23.0570	198564	A/G	MODERATE	ribosomalRNAmethyltransferase-likeprotein
LpanUA.23.1550	684034	A/G	MODERATE	DHHCzincfingerdomain-likeprotein
LpanUA.24.0270	69043	T/C	MODERATE	proteinphosphotase,putative
LpanUA.24.1120	384705	G/A	MODERATE	pre-mRNAsplicingfactor,putative
LpanUA.24.1450	502554	T/C	MODERATE	kinesin,putative
LpanUA.25.1710	809443	C/T	MODERATE	succinyl-CoAsynthetasealphasubunit,putative
LpanUA.25.2020	886263	C/T	MODERATE	metallo-peptidase,ClanMG,FamilyM24
LpanUA.25.2020	886286	C/T	MODERATE	metallo-peptidase,ClanMG,FamilyM24
LpanUA.25.2020	886364	G/A	MODERATE	metallo-peptidase,ClanMG,FamilyM24
LpanUA.25.2020	886427	G/A	MODERATE	metallo-peptidase,ClanMG,FamilyM24
LpanUA.25.2020	886470	C/A	MODERATE	metallo-peptidase,ClanMG,FamilyM24
LpanUA.25.2020	886568	G/T	MODERATE	metallo-peptidase,ClanMG,FamilyM24
LpanUA.25.2020	886593	T/C	MODERATE	metallo-peptidase,ClanMG,FamilyM24

LpanUA.26.0810	227095	A/G	MODERATE	glutathioneperoxidase-likeprotein,putative
LpanUA.27.0310	72890	G/A	MODERATE	methylmalonyl-coenzymeamutase,putative
LpanUA.28.2000	714017	A/G	MODERATE	DNArepairprotein-likeprotein
LpanUA.28.2160	771426	A/G	MODERATE	elongationfactorG2-likeprotein
LpanUA.28.2620	957492	G/A	MODERATE	2-oxoglutaratedehydrogenase,E2component,dihydrolipoamidesuccinyltransferase,putative
LpanUA.28.2880	1047672	A/G	MODERATE	proteinphosphatase4catalyticsubunit,putative
LpanUA.29.0080	19196	A/G	MODERATE	QA-SNAREproteinputative
LpanUA.29.0130	39634	T/C	MODERATE	proteasomeregulatorynon-ATPasesubunit,putative
LpanUA.29.0600	215973	A/G	MODERATE	DNArepairhelicase,putative
LpanUA.29.0800	281347	C/G	MODERATE	U-boxdomainprotein,putative
LpanUA.30.1410	424313	A/C	MODERATE	vacuolarassemblyproteinvps41,putative
LpanUA.30.1520	469483	C/T	MODERATE	ama1protein,putative
LpanUA.30.1520	469508	G/T	MODERATE	ama1protein,putative
LpanUA.30.1520	469576	T/C	MODERATE	ama1protein,putative
LpanUA.30.1520	469593	A/T	MODERATE	ama1protein,putative
LpanUA.30.1520	469604	G/T	MODERATE	ama1protein,putative
LpanUA.30.1520	469611	G/A	MODERATE	ama1protein,putative
LpanUA.30.1940	672132	A/G	MODERATE	cyclosomesubunit-likeprotein
LpanUA.31.0300	46190	C/T	MODERATE	nucleolarprotein,putative
LpanUA.31.0400	73423	A/G	MODERATE	mitoticcentromere-associatedkinesin MCAK ,putative
LpanUA.31.0590	129484	C/A	MODERATE	cysteinepeptidase,ClanCA,familyC2,putative
LpanUA.31.0590	131257	A/C	MODERATE	cysteinepeptidase,ClanCA,familyC2,putative
LpanUA.31.0760	195744	T/C	MODERATE	C-5steroldesaturase,putative
LpanUA.31.1110	312402	A/G	MODERATE	sodiumstibogluconateresistanceprotein,putative
LpanUA.31.1110	312435	G/A	MODERATE	sodiumstibogluconateresistanceprotein,putative
LpanUA.31.1650	583679	C/T	MODERATE	pentamidineresistanceprotein1
LpanUA.31.2020	758236	T/C	MODERATE	aminoacidpermease
LpanUA.31.2020	758260	A/C	MODERATE	aminoacidpermease
LpanUA.31.2020	758311	T/G	MODERATE	aminoacidpermease
LpanUA.31.1990	767733	C/T	MODERATE	aminoacidpermease
LpanUA.31.2260	865974	A/C	HIGH	metallo-peptidase,ClanMA M ,FamilyM8
LpanUA.31.2260	865980	T/C	MODERATE	metallo-peptidase,ClanMA M ,FamilyM8

LpanUA.31.3490	1309283	T/C	MODERATE	iron/zinctransporterprotein-likeprotein
LpanUA.31.3490	1309291	A/G	MODERATE	iron/zinctransporterprotein-likeprotein
LpanUA.31.3490	1310023	C/G	MODERATE	iron/zinctransporterprotein-likeprotein
LpanUA.32.0045	41	T/A	MODERATE	hypotheticalgamma-glutamyltransferase1hybridprotein
LpanUA.32.0045	68	G/A	MODERATE	hypotheticalgamma-glutamyltransferase1hybridprotein
LpanUA.32.1120	398367	T/C	MODERATE	proteinkinase,putative
LpanUA.33.0960	301760	G/T	MODERATE	beta-tubulin
LpanUA.33.0960	301784	A/G	MODERATE	beta-tubulin
LpanUA.33.0960	301785	C/T	MODERATE	beta-tubulin
LpanUA.33.0960	302143	T/G	MODERATE	beta-tubulin
LpanUA.33.0960	302150	C/A	MODERATE	beta-tubulin
LpanUA.33.0960	302182	G/T	MODERATE	beta-tubulin
LpanUA.33.0960	302546	G/T	MODERATE	beta-tubulin
LpanUA.33.1020	306874	A/G	MODERATE	2,4-dienoyl-coareductasefadh1,putative
LpanUA.33.1190	366142	T/C	MODERATE	protein,unknownfunction
LpanUA.33.1610	508635	A/G	MODERATE	QA-SNAREproteinputative
LpanUA.33.1730	604890	T/A	MODERATE	lipaseprecursor-likeprotein
LpanUA.33.3520	1431137	T/C	MODERATE	h1histone-likeprotein
LpanUA.34.0060	2379	C/T	MODERATE	pyruvatekinase
LpanUA.34.0060	2385	C/T	MODERATE	pyruvatekinase
LpanUA.34.0060	2475	A/G	MODERATE	pyruvatekinase
LpanUA.34.0060	2515	T/G	MODERATE	pyruvatekinase
LpanUA.34.0060	2549	C/T	MODERATE	pyruvatekinase
LpanUA.34.0060	2598	A/G	MODERATE	pyruvatekinase
LpanUA.34.0060	2618	G/A	MODERATE	pyruvatekinase
LpanUA.34.0060	3036	C/A	HIGH	pyruvatekinase
LpanUA.34.0060	3047	C/T	MODERATE	pyruvatekinase
LpanUA.34.0060	3137	T/C	MODERATE	pyruvatekinase
LpanUA.34.1060	441433	T/A	MODERATE	oligosaccharyltransferase-likeprotein
LpanUA.34.2270	873101	A/G	MODERATE	proteinkinase,putative
LpanUA.34.2300	886939	C/G	MODERATE	metallo-peptidase,ClanMG,FamilyM24
LpanUA.34.2370	923424	G/A	MODERATE	phosphoinositide-bindingprotein,putative
LpanUA.34.2970	1164392	C/T	MODERATE	ubiquitin-activatingenzymee1,putative

LpanUA.34.3010	1186737	A/C	MODERATE	ATP-dependentRNAhelicase,putative
LpanUA.34.3010	1188500	A/C	MODERATE	ATP-dependentRNAhelicase,putative
LpanUA.34.3401	1329367	A/C	MODERATE	ABCtransporter,putative
LpanUA.34.3403	1335645	A/G	MODERATE	expression-siteassociated ESAG3 ,putative
LpanUA.34.3403	1335904	A/G	MODERATE	expression-siteassociated ESAG3 ,putative
LpanUA.34.3403	1336123	T/C	MODERATE	expression-siteassociated ESAG3 ,putative
LpanUA.34.3403	1336342	G/C	MODERATE	expression-siteassociated ESAG3 ,putative
LpanUA.34.3403	1336726	G/T	MODERATE	expression-siteassociated ESAG3 ,putative
LpanUA.34.3403	1336873	T/C	MODERATE	expression-siteassociated ESAG3 ,putative
LpanUA.34.3403	1336885	C/T	MODERATE	expression-siteassociated ESAG3 ,putative
LpanUA.34.3403	1336952	T/G	MODERATE	expression-siteassociated ESAG3 ,putative
LpanUA.34.3403	1336953	C/T	MODERATE	expression-siteassociated ESAG3 ,putative
LpanUA.34.3403	1337185	C/T	MODERATE	expression-siteassociated ESAG3 ,putative
LpanUA.34.3403	1337205	G/A	MODERATE	expression-siteassociated ESAG3 ,putative
LpanUA.34.3403	1337206	A/G	MODERATE	expression-siteassociated ESAG3 ,putative
LpanUA.34.3403	1337241	A/G	MODERATE	expression-siteassociated ESAG3 ,putative
LpanUA.34.3440	1354527	G/T	MODERATE	structuralmaintenanceofchromosome SMC familyprotein,putative
LpanUA.34.4340	1656840	T/C	MODERATE	DNApolymeraseepsiloncatalyticsubunit,putative
LpanUA.34.5325	1960884	G/C	MODERATE	cathepsin-likeprotein
LpanUA.35.0100	13168	A/G	MODERATE	L-ribulokinase,putative
LpanUA.35.0100	14242	C/T	MODERATE	L-ribulokinase,putative
LpanUA.35.0650	157904	G/A	MODERATE	ubiquitin-likeprotein,putative
LpanUA.35.1280	431997	C/T	MODERATE	endonuclease/exonuclease/phosphatase,putative
LpanUA.35.1330	446550	C/T	MODERATE	GPI10
LpanUA.35.1330	446551	T/C	MODERATE	GPI10
LpanUA.35.1330	446566	T/C	MODERATE	GPI10
LpanUA.35.1330	446609	C/G	MODERATE	GPI10
LpanUA.35.1330	447280	C/T	MODERATE	GPI10
LpanUA.35.1330	447453	C/T	MODERATE	GPI10
LpanUA.35.1330	447631	T/C	MODERATE	GPI10
LpanUA.35.1330	447667	C/T	MODERATE	GPI10
LpanUA.35.1330	447675	G/A	MODERATE	GPI10
LpanUA.35.1330	447702	T/C	MODERATE	GPI10

LpanUA.35.1330	447712	T/C	MODERATE	GPI10
LpanUA.35.3910	1375696	G/A	MODERATE	calmodulin,putative
LpanUA.35.4500	1574404	T/C	MODERATE	proteinkinase-likeprotein
LpanUA.35.4720	1684049	T/C	MODERATE	aminoacidtransporter,putative
LpanUA.35.5180	1852903	T/C	MODERATE	translationinitiationfactor2subunit,putative
LpanUA.35.6310	2245263	T/C	MODERATE	l6202.3-likeprotein
